# Probiotic in the prevention of ventilator-associated pneumonia in critically ill patients: evidence from meta-analysis and trial sequential analysis of randomized clinical trials

**DOI:** 10.1186/s12890-022-01965-5

**Published:** 2022-04-28

**Authors:** Yue-chen Sun, Chen-yi Wang, Hai-li Wang, Yao Yuan, Jian-hong Lu, Lei Zhong

**Affiliations:** 1 Department of Emergency, Huzhou Central Hospital, Affiliated Central Hospital HuZhou University, Huzhou, 313000 Zhejiang Province China; 2 Department of Intensive Care Unit, Ningbo Yinzhou No. 2 Hospital, Ningbo, 315000 Zhejiang Province China; 3 Department of Obstetrics and Gynecology, Huzhou Central Hospital, Affiliated Central Hospital HuZhou University, Huzhou, 313000 Zhejiang Province China; 4 Department of Intensive Care Unit, Huzhou Central Hospital, Affiliated Central Hospital HuZhou University, No. 1558, North Sanhuan Road, Huzhou, 313000 Zhejiang Province China

**Keywords:** Critically ill patients, Probiotic, Prebiotic, Synbiotic, Ventilator-associated pneumonia

## Abstract

**Background:**

Probiotic might have a role in the prevention of ventilator-associated pneumonia (VAP) among mechanically ventilated patients, but the efficacy and safety remained inconsistent. The aim of this systematic review and meta-analysis was to evaluate the efficacy and safety of probiotic (prebiotic, synbiotic) versus placebo in preventing VAP in critically ill patients undergoing mechanical ventilation.

**Methods:**

PubMed, Embase and the Cochrane library databases were searched to 10 October 2021 without language restriction for randomized or semi-randomized controlled trials evaluating probiotic (prebiotic, synbiotic) vs. placebo in prevention of VAP in critically ill mechanically ventilated patients. The pooled relative risk (RR) along with 95% confidence intervals (CI) were combined using a random-effects model. Furthermore, the trial sequential analysis (TSA) and subgroup analyses were performed. Statistical significance was regarded as *P* < 0.05.

**Results:**

Twenty-three trials involving 5543 patients were eligible for this meta-analysis. The combined RR of decreasing the risk of VAP by probiotic was 0.67 (0.56, 0.81) for all eligible studies, 0.69 (n = 5136; 95% CI = 0.57 to 0.84; *P* < 0.01) for adults studies and 0.55 (n = 407; 95%CI = 0.31 to 0.99; *P* = 0.046) for neonates/children studies. Additionally, the above-mentioned positive finding in 20 adults studies was verified by the results of TSA, subgroup analyses and cumulative meta-analysis. Ample evidences demonstrated a 31% decrease in RR of incidence of VAP was noted when prophylactic probiotic therapy was administrated among adult patients. Finally, there were no effects on the ICU/hospital/28-/90-day mortality, bacteremia, CRBSI, diarrhea, ICU-acquired infections, infectious complications, pneumonia, UTI and wound infection between two groups (*P* > 0.05 for all).

**Conclusions:**

Based on the results of our study, the current evidences suggested that prophylactic administration of probiotic might be utilized as a preventive method for VAP in neonates/children and adults patients who required mechanical ventilation. However, further large, high-quality RCTs are warranted to assess the efficacy and safety of probiotic treatment in critically ill patients, especially for the neonates/children studies and the long-term consequences of this therapy.

**Supplementary Information:**

The online version contains supplementary material available at 10.1186/s12890-022-01965-5.

## Introduction

Ventilator-associated pneumonia (VAP), characterized as a type of nosocomial pneumonia that occurs at least 48 h after the initiation of mechanical ventilation (MV) in intensive care unit (ICU), leaded to prolonged the duration of MV, stay in ICU and hospital, as well as increased mortality and healthcare burden [1–3]. The latest data, conducted in 538,600 patients from 14 countries, revealed that the pooled incidence of VAP is 15.1 per 1000 ventilator-days (VD), and high-income countries (9.0 per 1000 VD) is lower than lower- and upper-middle-income countries (18.5 and 15.2, per 1000 VD, respectively) [4]. Ferrer et al., in a review of the epidemiology of ICU-acquired pneumonia [5], have estimated that the all-cause mortality attributable to VAP ranged from 20 to 50% and the overall attributable mortality associated with VAP was approximately 13%. Furthermore, a lately data from Japan indicated that the average hospitalization costs for patients with VAP was $67,080, significantly higher than that those without VAP ($32,196) [6].

On account of the high incidence, severity and enormous burdens of VAP, ample studies have assessed various kinds of prevention strategies, including pharmacological and non-pharmacological interventions, to prevent VAP over the years [7]. Fortunately, the incidence of VAP has been steadily decreasing in recent years possibly due to the application of the ventilator bundles, such as hand hygiene, oral care, semi-recumbent position, and subglottic secretion drainage system, daily sedation vacations as well as deep vein thrombosis prophylaxis, etc. [5, 7]. For quite a long time, antibiotic use has been the cornerstone of preventing and treating various infections, especially in ICU, but equally, inappropriate antimicrobial therapy was linked to potential risks such as affecting the microbiota composition, bringing the problem of superinfections and increasing the occurrence of drug-resistance bacteria [8–10].

Therefore, an emerging number of studies has assessed the non-antibiotic approaches for the prevention of VAP in the last few years. The term “probiotics”, defined as live nonpathogenic microorganisms that exert a health benefit to the host later, [11] first appeared in 1974 [12] and it might represent a novel non-antibiotic intervention [13]. The beneficial effects of probiotics in the prevention of VAP were not yet entirely elucidated, perhaps via modulating intestinal microbiota, adjusting immune response, improving gut barrier function and suppressing pathogenic bacteria overgrowth,etc. [12, 14].

There were several studies in this area over the last few years since the first study of probiotic (prebiotic and synbiotic) in preventing VAP in mechanically ventilated critically ill patients was published. A series of studies showed unfavorable results with regard to the prevention of VAP by probiotics [15–18]; nonetheless, other studies [19, 20] reported promising results, which were further confirmed by several meta-analyses [21–28].

Considering these controversial results, we therefore undertook a systematic review and meta-analysis to compare the efficacy and safety of administering probiotic (prebiotic, synbiotic) versus placebo on the prevention of VAP in critically ill ventilated patients.

## Methods

This study was written following the preferred reporting items for systematic reviews and meta-analyses (PRISMA) guidelines (Additional file 1: Appendix 1) [29].

### Search strategy and selection criteria

The clinical questions were specified using the PICO framework listed in Additional file 2: Appendix 2. Two writers (ZL and SYC) independently searched the PubMed, Embase and the Cochrane library databases to identify randomized controlled trials (RCTs) or quasi-RCTs that addressed the efficacy of probiotic, prebiotic or symbiotic supplementation in preventing VAP among critically ill patients from the inception to 10 October 2021, without language restriction. The keywords were as follows: “probiotic”, “prebiotic”, “synbiotic”, “ventilator-associated pneumonia”, “Randomized Controlled Trial”, etc. The Additional file 2: Appendix 2 provided a full description of the search strategy. Moreover, the reference lists of relevant papers were selectively hand-searched to capture any additional studies.

We excluded studies if they were duplicate publications, case reports, letters, reviews, case–control studies, cohort studies or non-human studies. Trials eligibility were carried out by the two independent authors (ZL and SYC) through screening titles, abstracts and even reading the full text.

The primary outcome was as follows: the incidence of VAP; Secondary endpoints included: ICU/hospital/28-/90-day mortality, bacteremia, catheter-related bloodstream infection (CRBSI), diarrhea, ICU-acquired infections, infectious complications, pneumonia, urinary tract infection (UTI) and wound infection.

### Data extraction

The relevant data of included articles were extracted by two separate authors (ZL and SYC) and were summarized in Table 1. We contacted original authors to ask for any relevant missing information whenever possible, for example, the Mahmoodpoor study [30].

**Table 1:** Core characteristics of the included studies

Study ID	Country origin	Sample size	Mean age (years)	Female (%)	APACHE II score	Follow-up time (day)	Treatment intervention	Control intervention	Center	Setting	Disease types
*Adult*											
Spindler-Vesel 2007 [50]	Slovenia	26/87	41.00	22.12	13.00	ICU stay	Synbiotic, qd	Control	S	SICU	Multiple injures
Forestier 2008 [17]	France	102/106	58.47	29.81	44.40^b^	78.00	Probiotic, bid	Placebo	S	ICU	Multi-disease
Knight 2009 [38]	England	130/129	49.75	37.84	17.00	Hospital stay	Synbiotic, bid	Placebo	S	ICU	Multi-disease
Giamarellos-Bourboulis 2009 [51]	Greece	36/36	54.40	18.46	19.36	28.00	Synbiotic, qd	Placebo	M	SICUs	Multiple injures
Morrow 2010 [20]	America	73/73	53.55	41.10	23.20	25.00	Probiotic, bid	Placebo	S	ICU	Multi-disease
Barraud 2010 [37]	France	87/80	60.70	59.28	59.80^b^	90.00	Probiotic, qd	Placebo	S	ICU	Multi-disease
Tan 2011 [34]	China	26/26	40.65	23.08	14.55	28.00	Probiotic, tid	Control	S	ICU	Severe traumatic brain-injured
Oudhuis 2011 [35]	Netherlands	130/124	62.72	38.19	22.02	75.00	Probiotic, bid	Control	M	ICUs	Multi-disease
Rongrungruang 2015 [39]	Thailand	75/75	71.02	58.67	19.65	90.00	Probiotic, qd	Control	S	ICU	Multi-disease
Zeng 2016 [36]	China	118/117	52.39	41.28	15.65	14.00	Probiotic, tid	Control	M	ICUs	Multi-disease
Zarinfar 2016 [41]	Iran	33/33	47.80	31.67	–	–	Probiotic, tid	Control	S	ICU	Multi-disease
Shimizu 2018 [19]	Japan	35/37	74.00	34.72	19.51	28.00	Synbiotic, qd	Control	M	ICUs	Sepsis
Klarin 2018 [52]	Sweden	69/68	65.75	44.53	22.99	180.00	Probiotic,bid	Control	M	ICUs	Multi-disease
Kooshki 2018 [53]	Iran	30/30	56.95	–	23.20	84.00	Prebiotic, bid^c^	Control	M	ICUs	Multi-disease
Anandaraj 2019 [54]	India	72/74	42.51	41.78	19.49	Hospital stay	Probiotic, bid	Placebo	S	ICU, HDU	Multi-disease
Mahmoodpoor 2019 [30]	Iran	48/54	58.25	46.08	23.41	14.00	Probiotic, bid	Placebo	M	SICUs	Multi-disease
Tsaousi 2019 [55]	Greece	28/30	–	–	–	30.00	Probiotic, qd	Placebo	S	ICU	Multiple injures
Habib 2020 [56]	Egypt	32/33	39.48	20.00	–	ICU stay	Probiotic, tid	Placebo	S	ICU	Multiple injures
Nazari 2020 [57]	Iran	73/74	52.60	31.29	–	Hospital stay	Probiotic,bid	Placebo	M	NICU	Multitrauma
Johnstone 2021 [18]	multicountry	1318/1332	59.85	40.11	22.00	60	Probiotic,bid	Placebo	M	ICUs	Multi-disease
*Neonates/children*											
Li 2012 [58]	China	82/83	0.61	44.24	–	–	Probiotic,qd	Control	S	PICU	Multi-disease
Banupriya 2015 [59]	India	75/75	2.92	39.33	11.43^a^	Hospital stay	Probiotic,bid	Control	S	PICU	Multi-disease
Angurana 2018 [40]	India	50/50	3.65	40.00	16.00	ICU stay	Probiotic, bid	Placebo	S	PICU	Severe Sepsis

APACHE II score, Acute Physiology and Chronic Health Evaluation II score; HDU, high dependency unit; ICU, intensive care unit; M multi-center study; NICU neurosurgical intensive care unit; PICU, pediatric intensive care unit

^a^PRISM score, Pediatric risk of mortality score; SICU, surgical ICU; S single-center study

^b^SAPS II score, Simplified acute physiology score II score

^c^Fenugreek seeds act as a prebiotic

### Assessment of study quality

We evaluated the quality of each eligible studies in adherence to The Cochrane Collaboration's tool [31], including selection bias, performance bias, detection bias, attrition bias, reporting bias, and other bias. Simultaneously, the strength of evidence for all outcomes in adults studies was assessed using GRADE (Grading of Recommendations Assessment, Development and Evaluation) methodology.

### Sensitivity analysis and publication bias

We did a sensitivity analysis so as to appraise the stability of the pooled effect estimates. The publication bias was also examined by the two authors via the funnel plot and statistical tests (Begg’s Test and Egger’s Test) [31]. In addition, we conducted a trim and fill analysis.

### Statistical analysis

For each trial, the dichotomous outcomes were reported as relative risk (RR) along with 95% confidence interval (CI). The median and range/interquartile range were converted to mean and standard deviation using the formulas described by one previous study [32]. The between-study heterogeneity was determined in accordance with the Chi-square test, *P* values and the *I*^*2*^ index. In view of the conservative of random-effects model, we used this model to pool all data [33]. In order to determine whether the accumulated evidence was sufficient and conclusive, a trial sequential analysis (TSA) was performed in our study. The TSA version 0.9.5.10 beta (www.ctu.dk/tsa), Stata 12.0 (StataCorp, College Station, TX, USA) and Review Manager Version 5.3.5 software (http://tech.cochrane.org/revman/download) were implemented to analyze data. A two-tailed *P*-value < 0.05 was considered statistically significant.

Probiotics, prebiotics and synbiotics were equal for analysis in our meta-analysis.

Considering the difference in neonates/children and adults, we analyzed the data separately. In addition, we conducted subgroup analyses based on the strain types (prebiotic vs. synbiotic vs. probiotic), the risk of bias (low risk vs high risk) and the center (multi-center vs. single-center). We also applied a cumulative meta-analysis by publication year.

## Results

### Search results and study characteristics

**Fig. 1:**
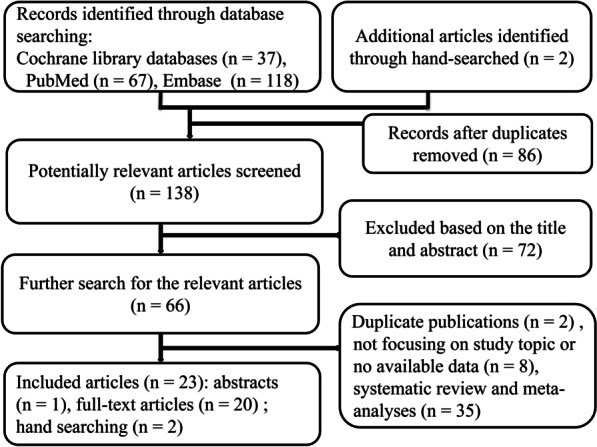
Flow diagram of study selection process

The chart of the study-selection procedure was presented in Fig. 1. Up to 10 October, 2021, 222 citations through the initial search of electronic databases were identified, and only 23 remnant trials, including 20 adult and 3 neonate/child populations studies, were ultimately included in our study. The 23 literatures, including 22 full-text articles and 1 abstract, on probiotics prophylaxis were reported between 2007 and 2021 and enrolled 52 to 2650 patients with a total of 5574 participants. The ages of the patients in adult populations ranged from 39.48 to 74.00 years. In studies eligible for inclusion in our meta-analysis, the follow-up times varied, ranging from 14 to 180 days with the proportion of female patients from 18.46 to 59.28%. Of them, the number of studies on patients treated with placebo/control compared to those treated with prebiotic, synbiotic and probiotic is 1, 4 and 18, respectively. Table 1 depicted the main characteristics of the 23 eligible trials.

### Assessment of study quality

**Fig. 2:**
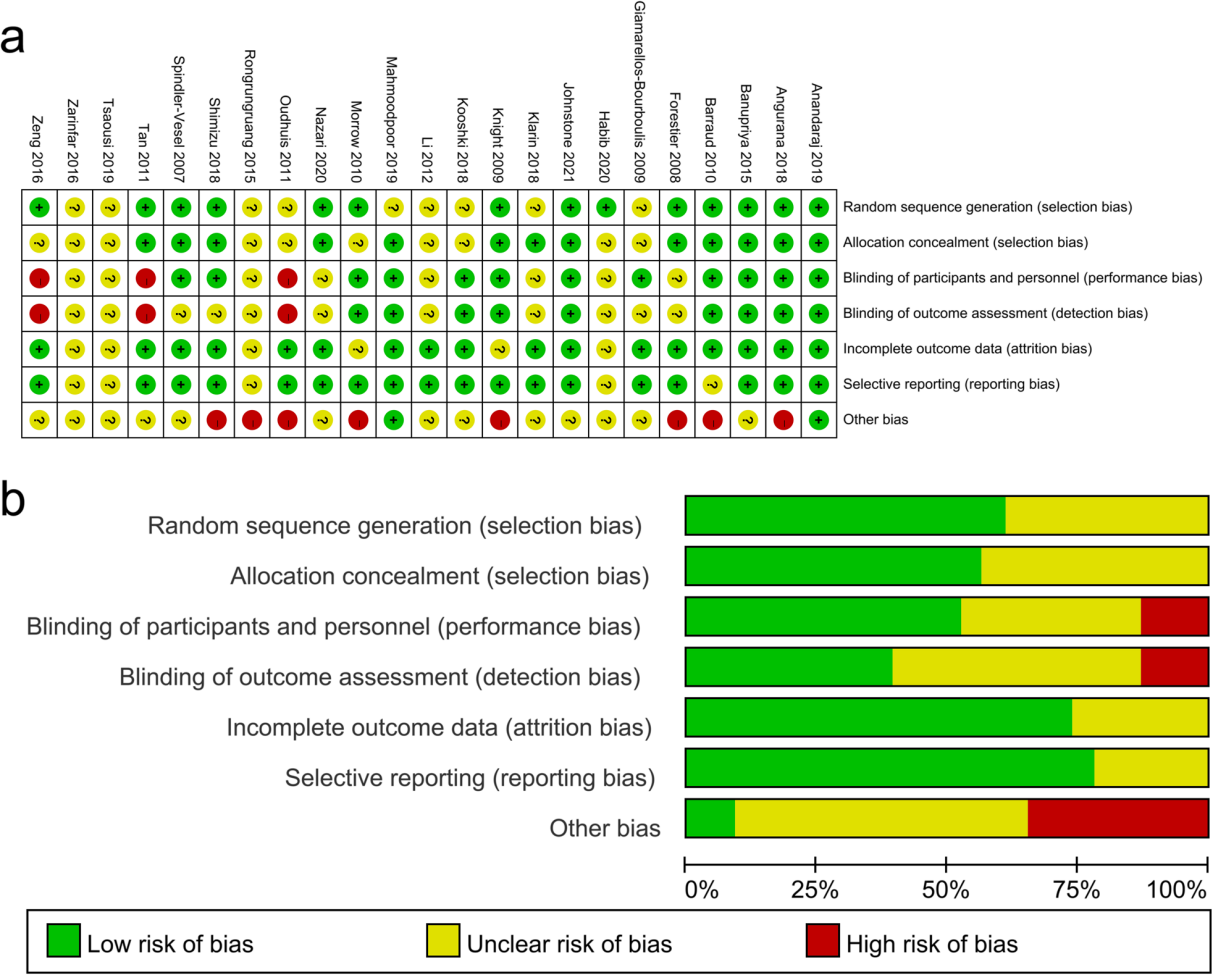
Risk of bias assessment. **a** Risks of bias summary. **b** Risks of bias graph

As listed in Fig. 2, a high risk of both performance and detection bias was presented in three studies [34–36] as a result of lacking of blinding or blind inadequacy. Because of a prematurely termination of schedule [35, 37], an imbalance in several significant baseline variables [20], an unreached of predetermined sample size [17, 19] and the funding provided by third parties [17, 20, 38–40], we rated these studies as having high risk of other bias. The quality of the evidence of probiotics in reducing VAP incidence in adult population was “high” (GRADE). Moreover, the quality of the evidence for secondary endpoints ranged from “very low” to “moderate” (Additional file 3: Appendix 3).

### Sensitivity analysis and assessment of reporting bias

The sensitivity analysis across studies for the primary outcome indicated the influence of each study set to the imputed RR is nonsignificant, demonstrating the stability of pooled estimate.

**Fig. 3:**
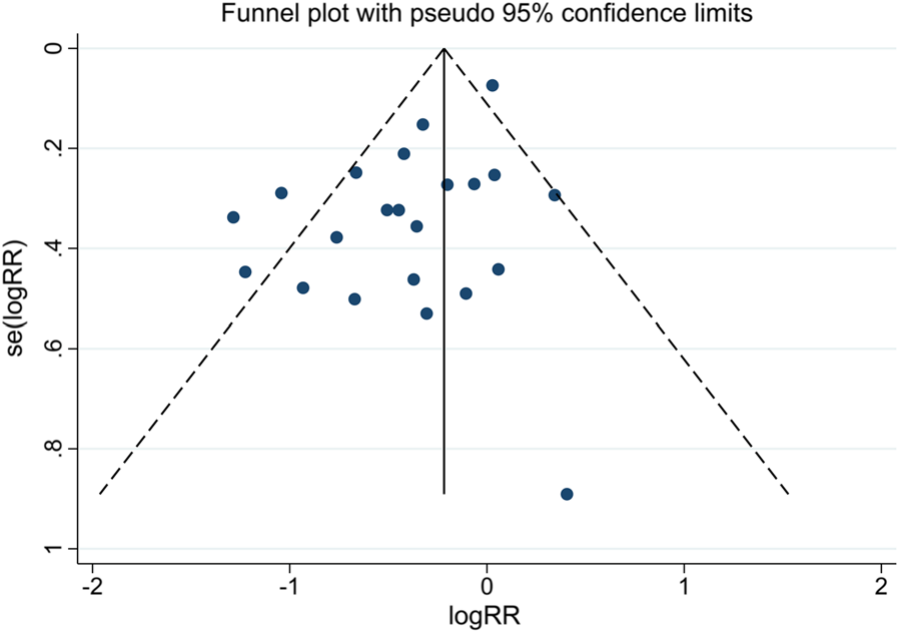
Funnel plot for publication bias. The blue dots and dotted line represent one single studies and 95% confidence intervals, respectively.

The publication bias existed by inspection of the funnel plot (Fig. 3), which was further confirmed through the Egger's test (*P* < 0.01). However, the Begg's test (*P* = 0.81) revealed no significant publication bias for our study. Then, a trim and fill method was used to identify potential publication bias, and the results showed that the impact of this bias is insignificant (Additional file 3: Appendix 3).

### Synthesis of primary outcome

**Fig. 4:**
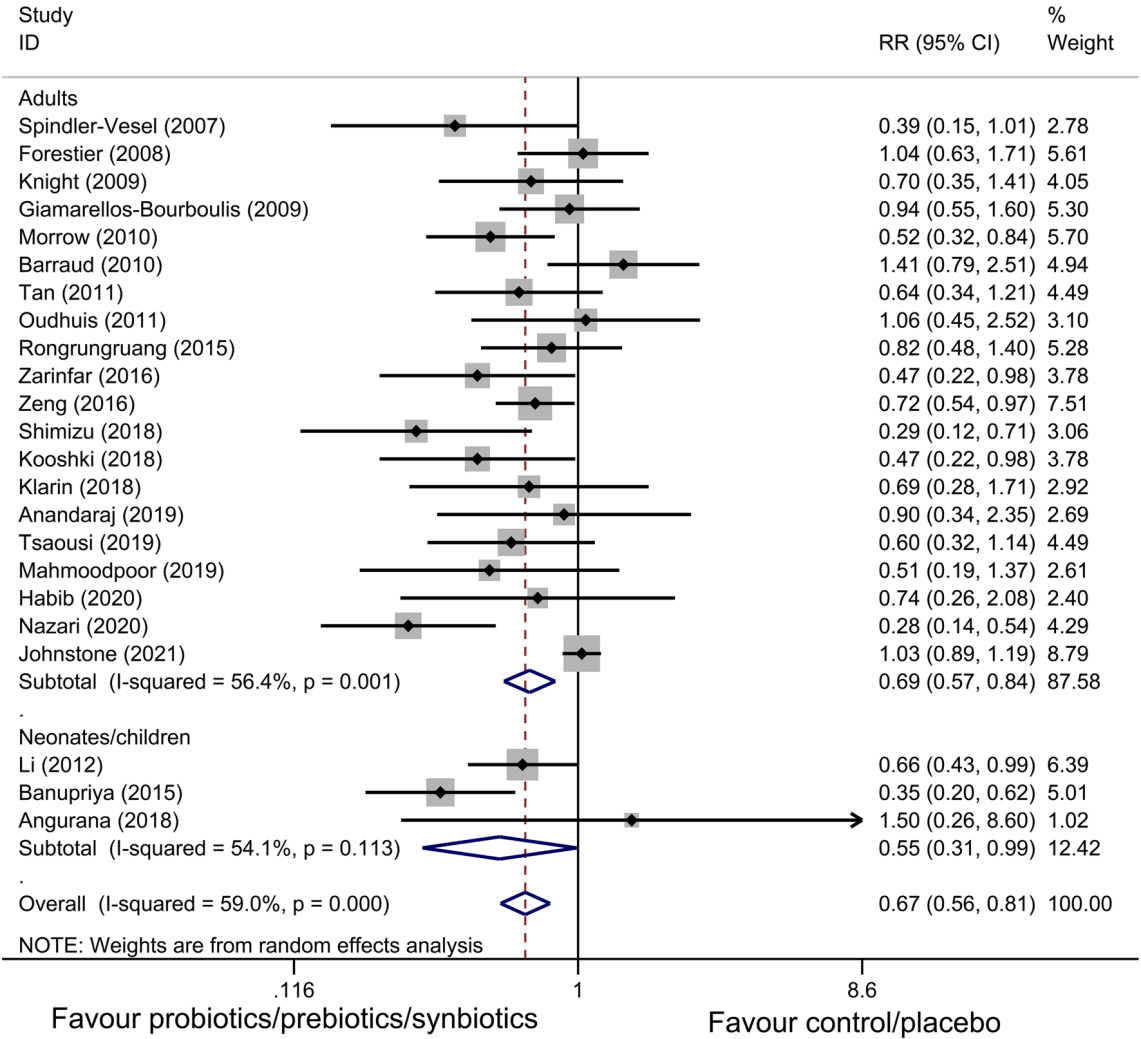
Forest plot of pooled data demonstrating the reduction in risk of ventilator-associated pneumonia incidence. RR relative risk, CI confidence interval

All 23 studies reported the main outcome of interest and the synthesized RR was 0.67 (n = 5543; 95% CI = 0.56 to 0.81; *P* < 0.01), with a moderate heterogeneity among these studies (*X*^*2*^ = 53.60, *P* < 0.01; *I*^*2*^ = 59.00%, Fig. 4). Meanwhile, the combined RR was 0.69 (n = 5136; 95% CI = 0.57 to 0.84; *P* < 0.05) for adults studies and 0.55 (n = 407; 95% CI = 0.31 to 0.99; *P* = 0.046) for neonates/children studies.

**Fig. 5:**
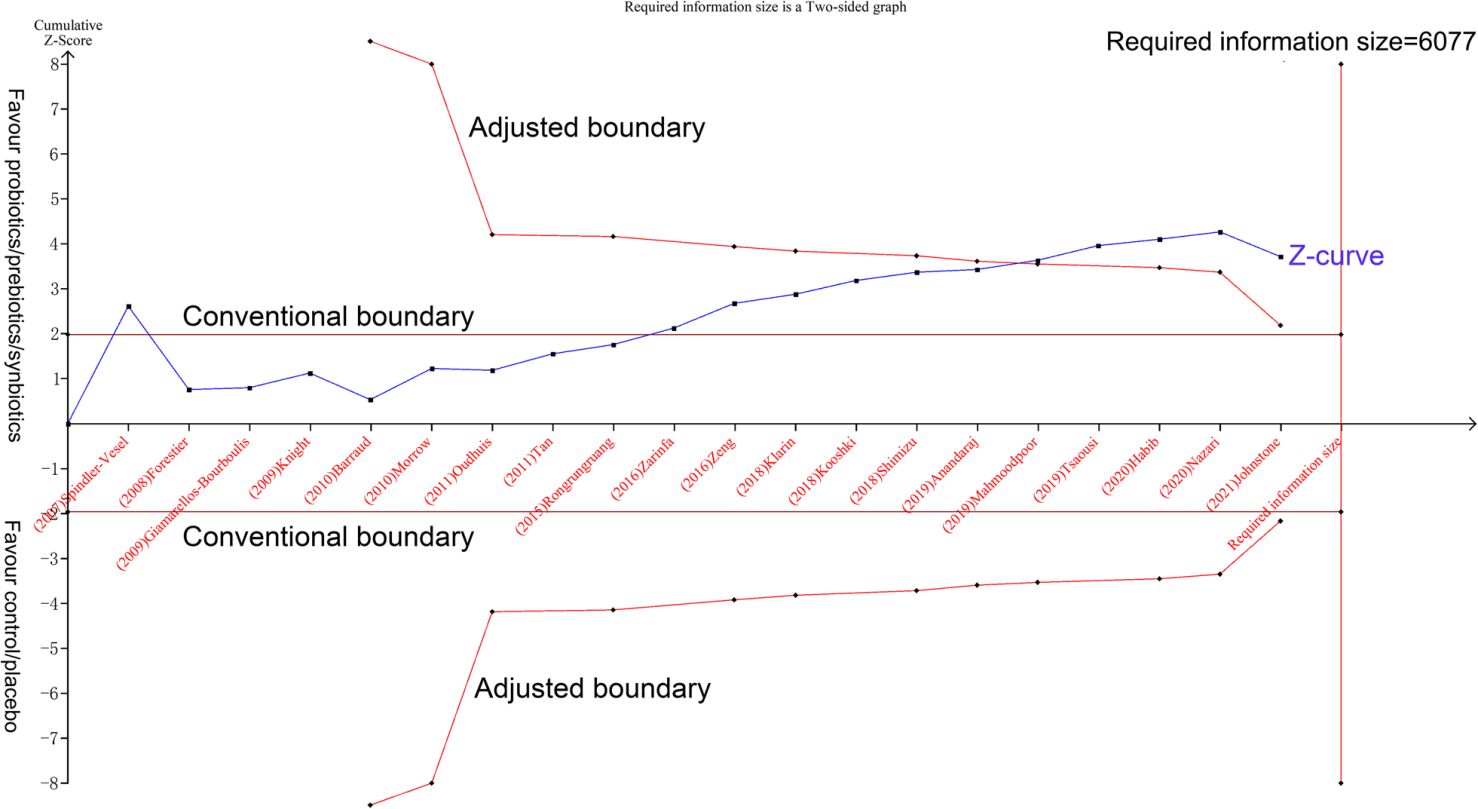
Trial sequential analysis for effects of probiotics on VAP incidence in adult patients. The required information size of 6077 was calculated based on the VAP incidence of 20.69, 25.27% in the probiotic and placebo group, respectively (α = 5%, β = 20%, *I*^*2*^ = 56.40%)

As shown in Fig. 5, although the accrued number of patients did not reach the required information size (RIS, 84.52%, 5136/6077), the cumulative Z-curve crossed the conventional boundary line and RIS-adjusted boundary value, thus indicating that a favorable effect of probiotic in preventing VAP is observed in adult patients. As revealed in Additional file 3: Appendix 3, however, the TSA of neonates/children patients showed that the cumulative Z-curve did not reach the adjusted boundary line and the optimal information size despite this line surpass the conventional boundary line slightly, indicating that the current evidence is inconclusive.

### Synthesis of secondary outcomes

Compared with the control (placebo) group, the probiotic (prebiotic, synbiotic) group had no significant effect on the ICU/hospital/28-/90-day mortality, bacteremia, CRBSI, diarrhea, ICU-acquired infections, infectious complications, pneumonia, UTI and wound infection (*P* > 0.05 for all, Additional file 3: Appendix 3).

### The results of subgroup analyses and cumulative meta-analysis in adult patients

From the prebiotic (n = 60; RR, 0.47; 95% CI = 0.22 to 0.98; *P* = 0.04), synbiotic (n = 516; RR, 0.57; 95% CI = 0.33 to 0.98; *P* = 0.04) and probiotic (n = 4560; RR, 0.74; 95% CI = 0.59 to 0.91; *P* = 0.01) analysis, the incidences of VAP in MV critically ill patients were proven to be significantly reduced by the use of this treatment. In subgroup analysis based on the risk of bias, a positive result was observed both in trials reporting low risk of bias (n = 3610; RR, 0.62; 95% CI = 0.45 to 0.85; *P* < 0.01) and in those reporting high risk of bias (n = 1526; RR, 0.76; 95% CI = 0.59 to 0.97; *P* = 0.03). This was also confirmed by another subgroup analysis of multi-center trials (n = 3729; RR, 0.64; 95% CI = 0.46 to 0.89; *P* = 0.01) versus single-center trials (n = 1407; RR, 0.73; 95% CI = 0.58 to 0.91; *P* = 0.01; Additional file 3: Appendix 3). Details of the results of this meta-analysis were shown in Table 2.

**Table 2:** Summary results on the primary outcome and subgroup analyses of this meta-analysis

Results	Primary outcome			Subgroup analyses (Adults, n = 20)						
	Total (n = 23)	Adults (n = 20)	neonates/children (n = 3)	Prebiotic (n = 1)	Symbiotic (n = 4)	Probiotic (n = 15)	low risk (n = 11)	high risk (n = 9)	multi-center (n = 9)	single-center (n = 11)
RR	0.67	0.69	0.55	0.47	0.57	0.74	0.62	0.76	0.64	0.73
95% CI lower-bound	0.56	0.57	0.31	0.22	0.33	0.59	0.45	0.59	0.46	0.58
95% CI upper-bound	0.81	0.84	0.99	0.98	0.98	0.91	0.85	0.97	0.89	0.91
*P*-value	< 0.01	< 0.01	0.046	0.04	0.04	0.01	< 0.01	0.03	0.01	0.01

RR relative risk, CI confidence interval

**Fig. 6:**
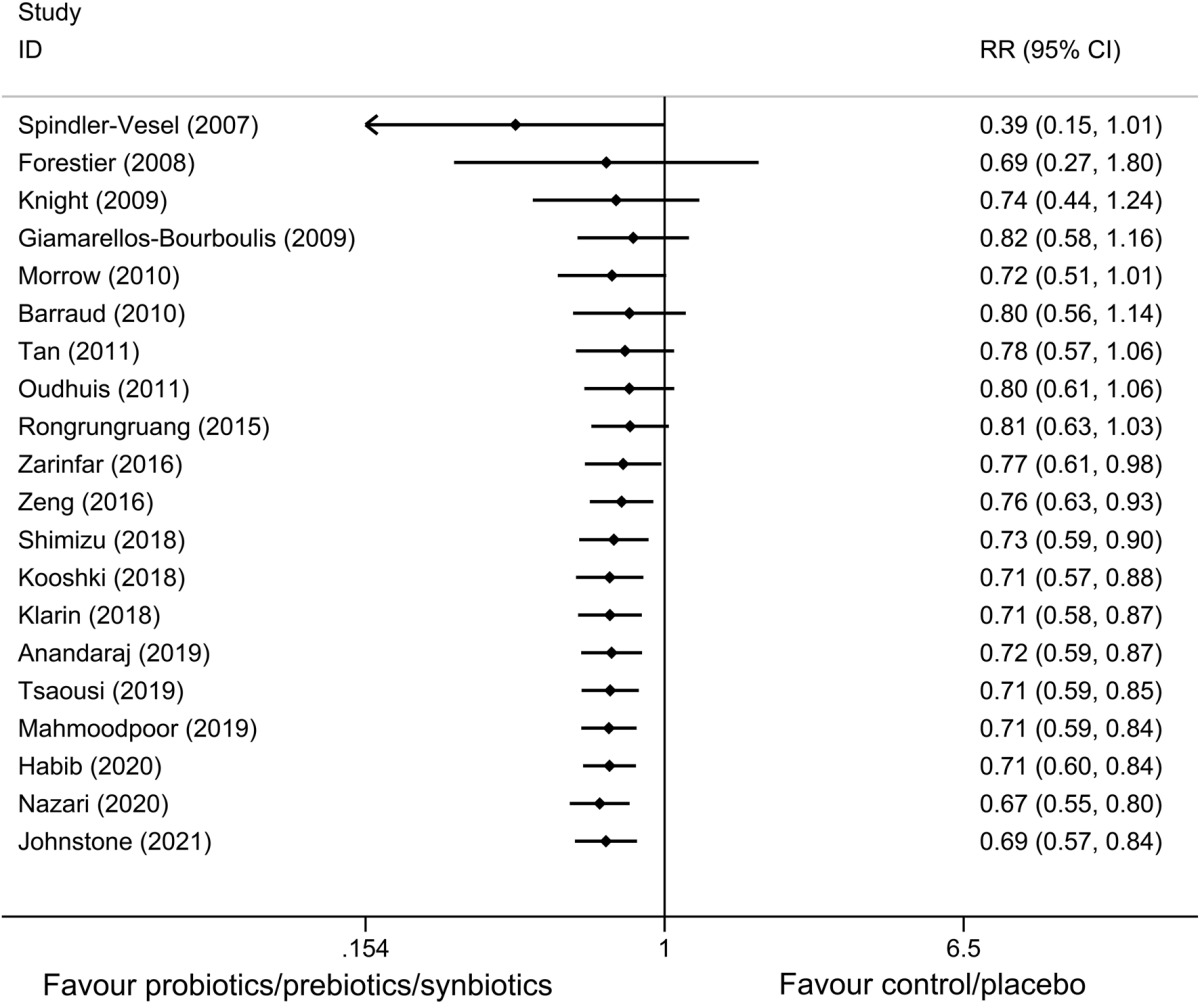
Cumulative meta-analysis showing the cumulative evidence of the efficacy of probiotic in preventing VAP in adult critically ill patients undergoing MV. VAP ventilator-associated pneumonia, MV mechanical ventilation, RR relative risk, CI confidence interval

Although no statistical significance that prophylactic probiotic among adult patients could result in a reduction of VAP incidence could be achieved before 2016 Zarinfar [41] studies showed a consistently positive result thereafter (Fig. 6).

## Discussion

The present systematic review and meta-analysis of 23 studies examined the effects of probiotic versus placebo in preventing VAP among critically ill patients and concluded that prophylactic probiotic therapy impacts positively on the incidence of VAP, with a 31%, 45% reduced risk in adults and neonates/children, respectively. Furthermore, the above mentioned positive result in adults patients was lately confirmed by the result of TSA, subgroup analyses and cumulative meta-analysis. There was no statistical difference of ICU/hospital/28-/90-day mortality, bacteremia, CRBSI, diarrhea, ICU-acquired infections, infectious complications, pneumonia, UTI and wound infection between two groups.

Diminishing the occurrence of VAP remains a challenge. Unlike previous recognition that lung is a sterile organ [42], there exists a “lung microbiota” in our lung. In healthy lungs, a dynamic balance between immigration of microorganism from the upper respiratory tract and elimination of bacteria by host defense mechanisms is existed [8]. Unfortunately, this balance is being disrupted when people suffer from several certain respiratory diseases, such as asthma, cystic fibrosis and lung infections, etc. [43]. Of note, the disruption of microbial homeostasis might be associated with the occurrence of VAP. Indeed, orotracheal intubation, which might impair the natural lung defense mechanisms, is a promoter of microbiome dysbiosis [44]. Furthermore, the gut–lung–microbiome axis is one of current researching hotspots in basic research in recent years. Significantly, this axis is bidirectional—gut dysbiosis is related to lung disorders and infections, whereas, the changes in lung microbial composition can affect the intestinal flora—mainly through the circulation of soluble microbial components and metabolites (ie, peptidoglycans, lipopolysaccharide) [8]. The source of bacterial dysbiosis in the lung might be derived from the gut, thus resulting in the occurrence of VAP [45]. Hence, we suspect that as a potential benefit of inhaled antibiotics in preventing VAP, “aerosolized probiotics” [46] might emerge in the near future, which may play a role in regulation of lung microbiome directly.

For quite a long time, probiotics are generally recognized as safe, and probiotic products are now ubiquitous in our lives, such as yogurts, cheeses, snacks and cosmetic products, etc. [12]. Moreover, probiotics are increasingly given as accessory or therapeutic method to hospitalized patients, especially for the critically ill patients (eg. VAP, sepsis and antibiotic-associated diarrhea, etc.) [47]. Despite probiotic products and probiotics are being used widely in life and clinical practice, their safety has not been fully assessed. Recently, some of scholars have expressed their concern as regards the probiotic safety [11, 48] and Nieuwboer, et al. [47] suggested that a solid evidence for the proper and safe use of probiotics is still needed to be established, in particular for high-risk population (eg. prematurity, immunocompromised and critically ill patients, etc.). Conversely, Cabana and colleagues [49] reported that some of probiotic strains were subject to stringent safety evaluation followed by notification of the US Food and Drug Administration for comment, and the data from many high-quality studies have tracked adverse complications and provided evidences in favor of probiotics. In our meta-analysis, 8.70% (2/23) of the eligible studies expressed a degree of uncertainty about the safety, 17.39% (4/23) of the studies were silent about the safety issues, and 69.57% (16/23) of the studies have indicated that no obvious adverse events attributed to the probiotic (prebiotic, synbiotic) were noted in these study populations. Nonetheless, a large multicenter, randomized, concealed, blinded trial of 2650 critically ill patients (4.35%, 1/23) [18], found that compared with the placebo therapy, administration of the probiotic (lactobacillus rhamnosus GG) did not decrease the occurrence of VAP, and an increased risk of adverse events was noted among patients receiving this treatment.

There have been several relevant meta-analyses in this area to date, producing several conflicting outcomes [15, 16, 21–28]. Gu et al. [15] in 2012 published a meta-analysis of seven trials and failed to demonstrate a beneficial effect in reducing VAP in adult patients undergoing MV, and the result was further reinforced by a 2013 meta-analysis [16] with five trials. By contrast, an earlier meta-analysis [21] in 2010 concurred with our findings and revealed that the administration of probiotics is associated with a reduction in VAP incidence in adult patients who are mechanically ventilated, which was further proved by a subsequent 2014 Cochrane review with eight trials [22], two meta-analyses for adult and children patients [23, 24] and several meta-analyses for adult or (and) children patients [25–28]. Previous meta-analyses on this issue have focused on only adult patients or the combination analysis of both adult patients and non-adult patients.

The current meta-analysis has several strengths compared to earlier works. First, this study, to our knowledge, might be the first cumulative meta-analysis which conducted the TSA from the view of adult and neonates/children populations, resulting in a more robust, reliable and precise pooled estimate. Second, in contrast to prior meta-analyses, we analyzed the influences of probiotic on VAP from the viewpoint of neonates/children and adults populations, respectively, which was partly reflected a true effect of probiotic in the prevention of VAP in mechanically ventilated patients. Third, as the evidence accumulates and sample size increases, especially with the addition of a large new study (n = 2650) [18], our study had enhanced the statistical power to examine the efficacy of protective effects of probiotics in reducing VAP incidence.

Our meta-analysis has several potential shortcomings as well. First, since the possibility of false positive result in TSA, as well as the limited numbers of the eligible articles and samples, the positive result for neonates/children patients should be interpreted with caution. Consequently, further study on the beneficial effects of probiotics on VAP for these patients is needed. Second, the diagnosis of VAP might be complex due to the lack of uniformly accepted diagnostic standard, which might lead to increased the heterogeneity among these included studies. Finally, another limitation of the study is that it has not pre-registered a PROSPERO registration number. Thus, further large studies, especially for the neonates/children and an objective accepted diagnostic criteria of VAP, are necessary to verify our findings in this area.

## Conclusion

In conclusion, our cumulative meta-analysis strengthens the evidence that prophylactic use of probiotics may be a possible effective non-antibiotic option in reducing the incidence of VAP in critically ill ventilated patients. However, the long-run effects of probiotics safety on individuals warrant further studies, especially in special groups of critically ill patients (i.e. neonates/children, immunocompromised, severely debilitated patients, etc.).

## Supplementary Information

**Additional file 1. Appendix 1**: The PRISMA guidelines

**Additional file 2. Appendix 2**: The PICO framework, search strategy and search results

**Additional file 3. Appendix 3**: The results of sensitivity analysis, reporting bias, trial sequential analysis and forest plots

## Data Availability

All data are fully available without restriction.

## Abbreviations

CI: Confidence interval
CRBSI: Catheter-related bloodstream infection
HLOS: Hospital length of stay
ICU: Intensive care unit
MV: Mechanical ventilation
RCTs: Randomized controlled trials
RR: Relative risk
TSA: Trial sequential analysis
UTI: Urinary tract infection
VAP: Ventilator-associated pneumonia
VD: Ventilator-days
