# Supplementary material for: Probiotic in the prevention of ventilator-associated pneumonia in critically ill patients: evidence from meta-analysis and trial sequential analysis of randomized clinical trials
Source: BMC Pulm Med. 2022 Apr 28;22:168. doi: 10.1186/s12890-022-01965-5 (PMC9052689; doi:10.1186/s12890-022-01965-5)
Supplement: Supplementary file 2 — Additional file 2. Appendix 2: The PICO framework, search strategy and search results [file 12890_2022_1965_MOESM2_ESM.docx]

**Appendix 2: PICO framework**

| **PICO element Study inclusion criterion** |
| --- |
| **Patients** Critically ill mechanically ventilated patients  **Interventions** Probiotics, prebiotics, synbiotics  **Comparisons** Placebo or control  **Outcomes The primary endpoints**: the occurrence of VAP;  **The secondary endpoints**: ICU/hospital/28-/90-day mortality, bacteremia, catheter-related bloodstream infection, diarrhea, ICU-acquired infections, infectious complications, pneumonia, urinary tract infection and wound infection |

**The search strategy and search results**

PubMed (n=67), the Cochrane library databases(n=37) and Embase (n=118)

**PubMed:**

**#1** "Probiotics"[MeSH Terms] OR "Probiotics"[Title/Abstract] OR "probiotic*"[Title/Abstract] OR "Prebiotics"[MeSH Terms] OR "Prebiotics"[Title/Abstract] OR "prebiotic*"[Title/Abstract] OR "Synbiotics"[MeSH Terms] OR "Synbiotics"[Title/Abstract] OR "synbiotic*"[Title/Abstract] 40,314

**#2** (((("Pneumonia, Ventilator-Associated"[Mesh]) OR ("Ventilator-Associated Pneumonia"[Title/Abstract])) OR ("Pneumonia, Ventilator-Associated"[Title/Abstract])) OR ("Pneumonia, Ventilator Associated"[Title/Abstract])) OR ("Ventilator Associated Pneumonia"[Title/Abstract]) 6,816

**#3** "trial*"[Title/Abstract] OR "random*"[Title/Abstract] OR "Randomized Controlled Trial"[Publication Type] OR "Randomized Controlled Trials as Topic"[MeSH Terms] 2,040,590

**#4** #1 and #2 and #3 **67 (search results)**

**Cochrane library:**

Search Name: search202110101

Last Saved: 10/10/2021 12:30:48

ID Search

#1 ("Pneumonia, Ventilator-Associated"):ti,ab,kw OR ("Pneumonia, Ventilator Associated"):ti,ab,kw OR ("Ventilator-Associated Pneumonia"):ti,ab,kw OR ("Ventilator Associated Pneumonia"):ti,ab,kw (Word variations have been searched)

#2 MeSH descriptor: [Pneumonia, Ventilator-Associated] explode all trees

#3 #1 or #2

#4 ("Probiotics"):ti,ab,kw OR ("Probiotic"):ti,ab,kw OR ("Prebiotics"):ti,ab,kw OR ("Prebiotic"):ti,ab,kw OR ("Synbiotics"):ti,ab,kw (Word variations have been searched)

#5 ("Synbiotic"):ti,ab,kw OR ("probiotic*"):ti,ab,kw OR ("prebiotic*"):ti,ab,kw OR ("synbiotic*"):ti,ab,kw (Word variations have been searched)

#6 MeSH descriptor: [Probiotics] explode all trees

#7 MeSH descriptor: [Prebiotics] explode all trees

#8 MeSH descriptor: [Synbiotics] explode all trees

#9 #4 or #5 or #6 or #7 or #8

#10 ("trial*"):ti,ab,kw OR ("random*"):ti,ab,kw OR ("random"):ti,ab,kw OR ("randomized controlled trial"):ti,ab,kw (Word variations have been searched)

#11 MeSH descriptor: [Randomized Controlled Trial] explode all trees

#12 MeSH descriptor: [Randomized Controlled Trials as Topic] explode all trees

#13 #10 or #11 or #12

#14 #3 and #9 and #13 **37 (search results)**

**Embase Session Results (10 October 2021)** **（search results: 118）**

Embase

Session Results

.......................................................

No. Query Results Results Date

#25. #13 AND #19 AND #24 **118**  10 Oct 2021

#24. #20 OR #21 OR #22 OR #23 2,800,821 10 Oct 2021

#23. 'randomized controlled trial (topic)'/exp 212,134 10 Oct 2021

#22. 'randomized controlled trial'/exp 681,782 10 Oct 2021

#21. 'random*':ab,ti,kw 1,716,848 10 Oct 2021

#20. 'trial*':ab,ti,kw 1,651,048 10 Oct 2021

#19. #14 OR #15 OR #16 OR #17 OR #18 13,723 10 Oct 2021

#18. 'pneumonia, ventilator associated':ab,ti,kw 159 10 Oct 2021

#17. 'ventilator associated pneumonia':ab,ti,kw 9,124 10 Oct 2021

#16. 'ventilator-associated pneumonia':ab,ti,kw 9,128 10 Oct 2021

#15. 'pneumonia, ventilator-associated':ab,ti,kw 159 10 Oct 2021

#14. 'ventilator associated pneumonia'/exp 11,907 10 Oct 2021

#13. #1 OR #2 OR #3 OR #4 OR #5 OR #6 OR #7 OR #8 OR 58,478 10 Oct 2021

#9 OR #10 OR #11 OR #12

#12. 'synbiotic agent'/exp 2,177 10 Oct 2021

#11. 'prebiotic agent'/exp 573 10 Oct 2021

#10. 'probiotic agent'/exp 42,319 10 Oct 2021

#9. 'synbiotic*':ab,ti,kw 2,266 10 Oct 2021

#8. 'synbiotic':ab,ti,kw 1,387 10 Oct 2021

#7. 'synbiotics':ab,ti,kw 1,452 10 Oct 2021

#6. 'prebiotic*':ab,ti,kw 12,389 10 Oct 2021

#5. 'prebiotic':ab,ti,kw 8,437 10 Oct 2021

#4. 'prebiotics':ab,ti,kw 6,126 10 Oct 2021

#3. 'probiotic*':ab,ti,kw 38,428 10 Oct 2021

#2. 'probiotic':ab,ti,kw 25,165 10 Oct 2021

#1. 'probiotics':ab,ti,kw 26,464 10 Oct 2021
