# Supplementary material for: Probiotic in the prevention of ventilator-associated pneumonia in critically ill patients: evidence from meta-analysis and trial sequential analysis of randomized clinical trials
Source: BMC Pulm Med. 2022 Apr 28;22:168. doi: 10.1186/s12890-022-01965-5 (PMC9052689; doi:10.1186/s12890-022-01965-5)
Supplement: Supplementary file 3 — Additional file 3. Appendix 3: The results of sensitivity analysis, reporting bias, trial sequential analysis and forest plots [file 12890_2022_1965_MOESM3_ESM.docx]

**Appendix 3** The results of sensitivity analysis, reporting bias, trial sequential

analysis and forest plots.

**Sensitivity analysis**

**Assessment of reporting biases**

**Begg's Test**

adj. Kendall's Score (P-Q) = -10

Std. Dev. of Score = 37.86 (corrected for ties)

Number of Studies = 23

z = -0.26

Pr > |z| = 0.792

z = 0.24 (continuity corrected)

Pr > |z| = 0.812 (continuity corrected)

Egger's test

-------------------------------------------------------------------------------------------

Std_Eff | Coef. Std. Err. t P>|t| [95% Conf. Interval]

-------------+-----------------------------------------------------------------------------

slope| 0.0706373 0.1148345 0.62 0.545 -.1681742 0.3094488

bias| -1.484658 0.4864057 -3.05 0.006 -2.496194 -.4731217

-------------------------------------------------------------------------------------------

**Begg's Test**

**Egger's test**

**GRADE assessments for all outcomes** **in adults populations**

| **Outcome** | **Number（RCT）** | **Risk of bias** | **Inconsistency** | **Indirectness** | **Imprecision** | **Publication of bias** | **№ of patients**  **intervention contrast** | | **Effect size**  **（RR 95% CI）** | **Quality of evidence** |
| --- | --- | --- | --- | --- | --- | --- | --- | --- | --- | --- |
| **The incidence of VAP** | 20 | not serious | not serious | not serious | not serious | not serious | 523/2528（20.7%） | 659/2608（25.3%） | 0.69（0.57，0.84） | ⊕⊕⊕⊕  High |
| **ICU** **mortality** | 10 | not serious | not serious | not serious | serious ^d^ | not serious | 427/2030（21.0%） | 446/2098（21.3%） | 0.97（0.86，1.09） | ⊕⊕⊕〇  Moderate |
| **Hospital mortality** | 6 | not serious | not serious | not serious | serious ^d^ | not serious | 453/1722（26.3%） | 487/1741（28.0%） | 0.94(0.85,1.05) | ⊕⊕⊕〇  Moderate |
| **28-day mortality** | 6 | Serious ^abc^ | not serious | not serious | serious ^d^ | strongly suspected ^e^ | 54/287（18.8%） | 57/284（20.1%） | 0.94((0.67,1.31) | ⊕〇〇〇  Very low |
| **90-day mortality** | 2 | Serious ^bc^ | not serious | not serious | serious ^d^ | strongly suspected ^e^ | 52/162（32.1%） | 50/155（32.2%） | 1.00(0.72,1.37) | ⊕〇〇〇  Very low |
| **Bacteremia** | 3 | not serious | not serious | not serious | serious ^d^ | not serious | 113/1389（8.1%） | 115/1405（8.2%） | 0.79（0.37，1.70） | ⊕⊕⊕〇  Moderate |
| **catheter-related bloodstream infection** | 5 | serious ^a^ | not serious | not serious | serious ^d^ | not serious | 25/297（8.4%） | 41/347（11.8%） | 0.61(0.35,1.09) | ⊕⊕〇〇  Low |
| **diarrhea** | 7 | serious ^c^ | not serious | not serious | serious ^d^ | strongly suspected ^e^ | 1199/1756（68.3%） | 1208/1770（68.2%） | 1.00(0.95,1.07) | ⊕〇〇〇  Very low |
| **ICU-acquired infections** | 2 | serious ^b^ | not serious | not serious | serious ^d^ | not serious | 70/217（32.3%） | 60/204（29.4%） | 1.08(0.79,1.49) | ⊕⊕〇〇  Low |
| **Infectious complications** | 4 | serious ^a^ | not serious | not serious | serious ^d^ | not serious | 91/217（41.9%） | 128/274（46.7%） | 0.64(0.29,1.40) | ⊕⊕〇〇  Low |
| **Pneumonia** | 3 | serious ^a^ | not serious | not serious | serious ^d^ | not serious | 311/1458（21.3%） | 301/1462（20.6%） | 1.04(0.90,1.19) | ⊕⊕〇〇  Low |
| **Urinary tract infection (UTI)** | 6 | not serious | not serious | not serious | serious ^d^ | not serious | 211/1623（13.0%） | 200/1685（11.9%） | 1.10(0.57,2.14) | ⊕⊕⊕〇  Moderate |
| **Wound infection** | 3 | serious ^a^ | not serious | not serious | serious ^d^ | not serious | 3/182（1.6%） | 8/237（3.4%） | 0.65(0.17,2.52) | ⊕⊕〇〇  Low |

**Note :**a：lacking of blinding or blind inadequacy；b：prematurely termination of schedule; c：the funding provided by parties；d：The combined confidence intervals all included invalid lines；e ：Due to the existence of various financial support positive may increase.

**Trim-and-fill analysis**

Note: option 'ci' specified.

Meta-analysis

| Pooled 95% CI Asymptotic No. of

Method | Est Lower Upper z_value p_value studies

-------+----------------------------------------------------

Fixed | -0.217 -0.314 -0.121 -4.416 0.000 23

Random | -0.400 -0.587 -0.213 -4.188 0.000

Test for heterogeneity: Q= 53.573 on 22 degrees of freedom (p= 0.000)

Moment-based estimate of between studies variance = 0.098

Trimming estimator: Linear

Meta-analysis type: Random-effects model

iteration | estimate Tn # to trim diff

----------+--------------------------------------

1 | -0.400 136 0 276

2 | -0.400 136 0 0

Note: no trimming performed; data unchanged

Filled

Meta-analysis (exponential form)

| Pooled 95% CI Asymptotic No. of

Method | Est Lower Upper z_value p_value studies

-------+----------------------------------------------------

Fixed | 0.805 0.731 0.886 -4.416 0.000 23

Random | 0.671 0.556 0.808 -4.188 0.000

Test for heterogeneity: Q= 53.573 on 22 degrees of freedom (p= 0.000)

Moment-based estimate of between studies variance = 0.098

**Trial sequential analysis**

**
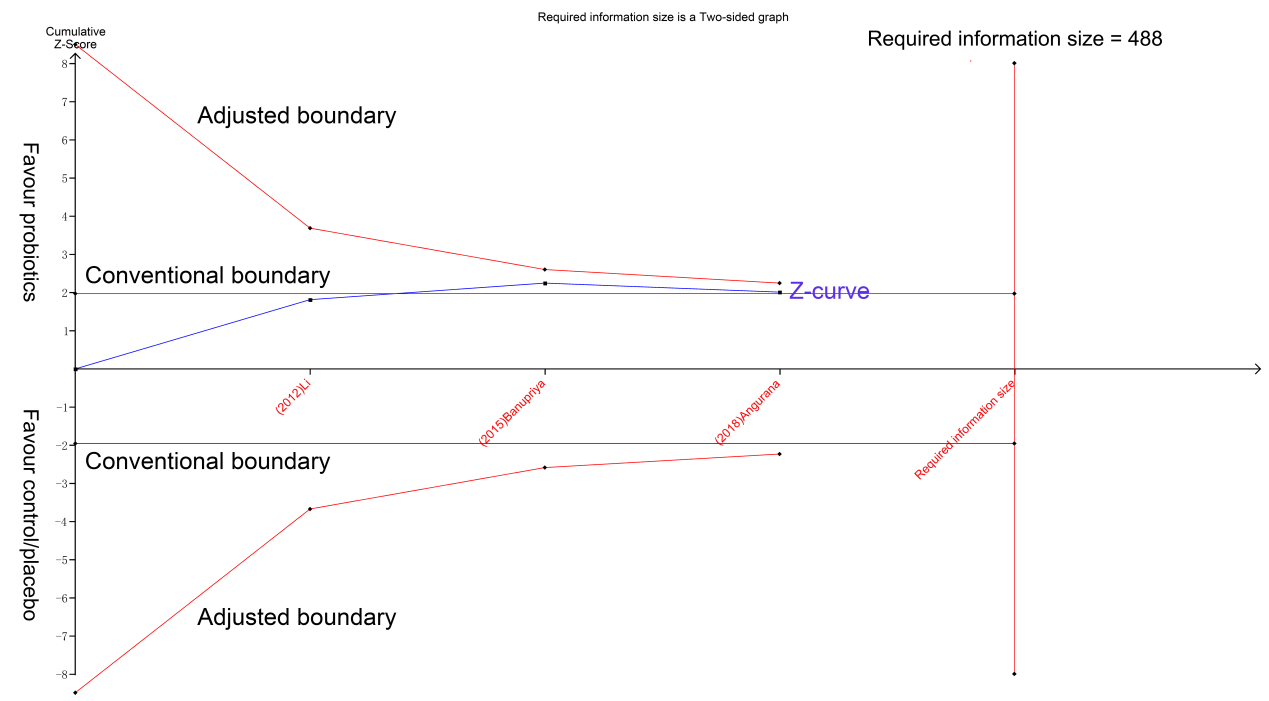
**

**Fig.** Trial sequential analysis for effects of probiotics on VAP incidence among neonates/children patients. The required information size of 488 was calculated based on the VAP incidence of 19.31, 36.10% in the probiotic and placebo group, respectively (α=5%, β=20%, *I^2^*=54.10%).

The trial sequential analysis result indicated that 407 (83.40%) of the required information size of 488 patients was accrued.

**Funnel plots**


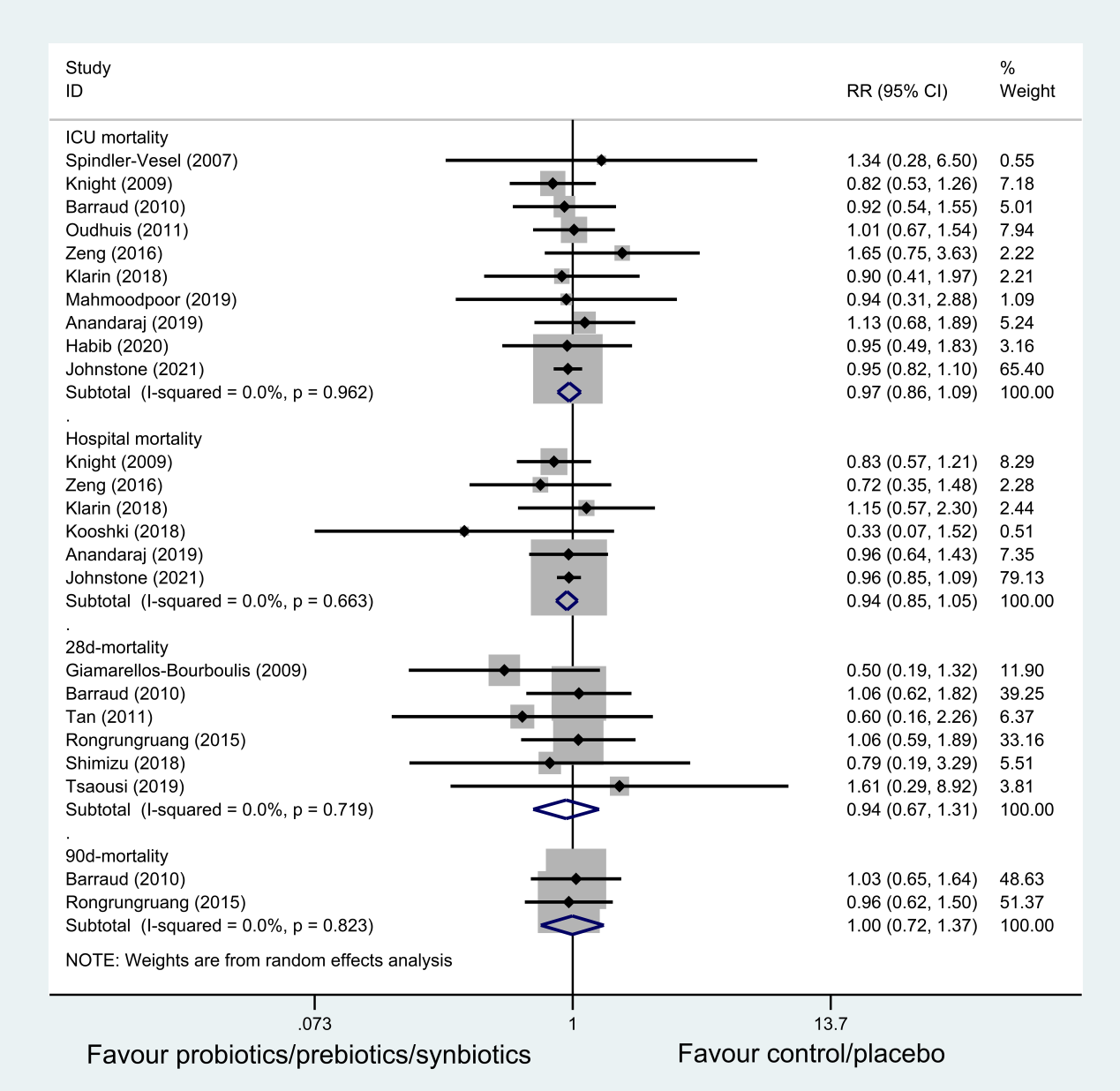


**a.** Forest plot of eligible studies evaluating effects of the probiotic (prebiotic, synbiotic) versus control (placebo) on ICU, hospital, 28d- and 90d-mortality. RR relative risk, CI confidence interval.


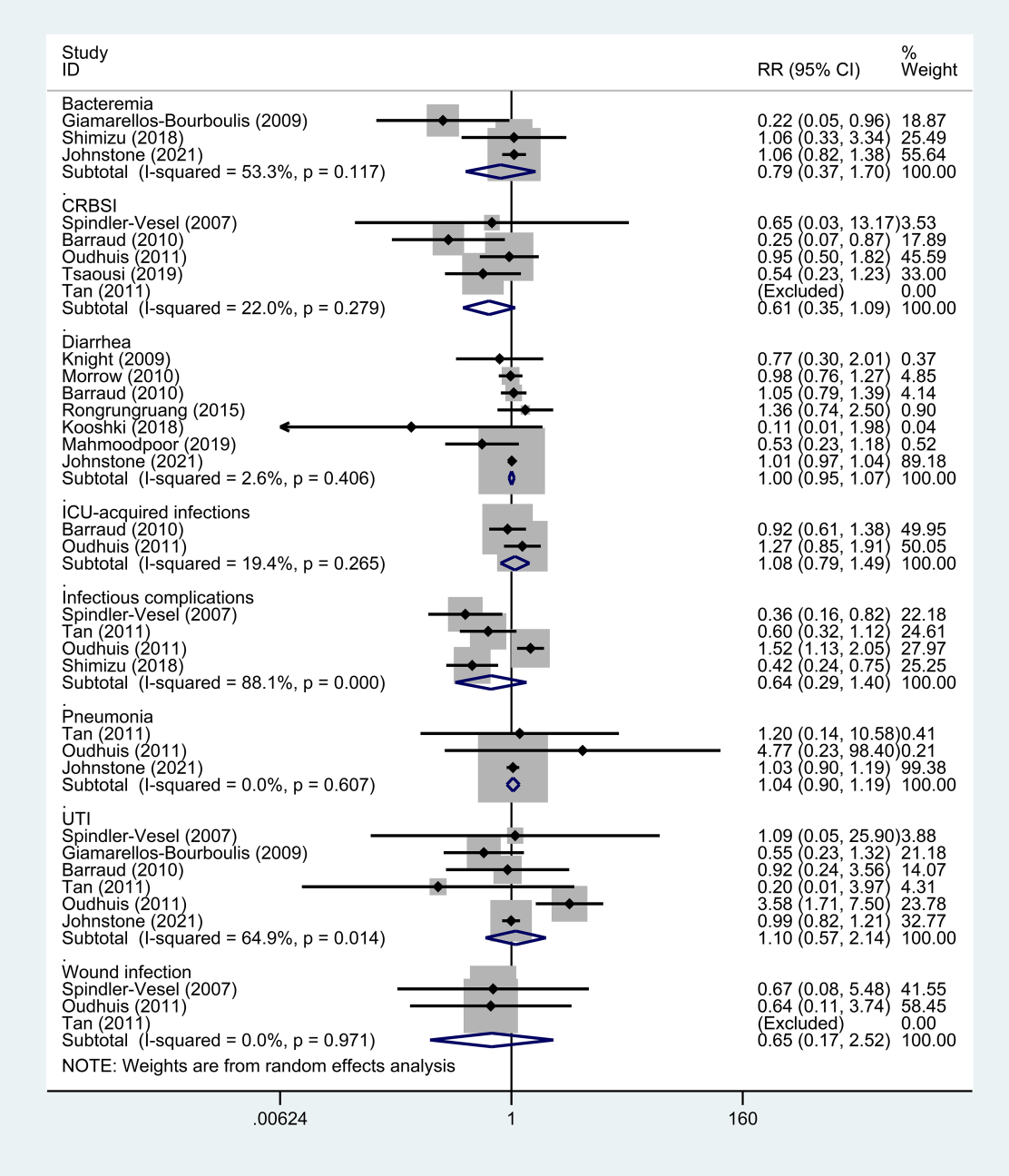


**b.** Forest plot of the probiotic (prebiotic, synbiotic) versus control (placebo) on the bacteremia, CRBSI, diarrhea, ICU-acquired infections, infectious complications, pneumonia, UTI and wound infection. ICU intensive care unit, CRBSI, catheter related blood stream infection, UTI, urinary tract infection, RR relative risk, CI confidence interval.


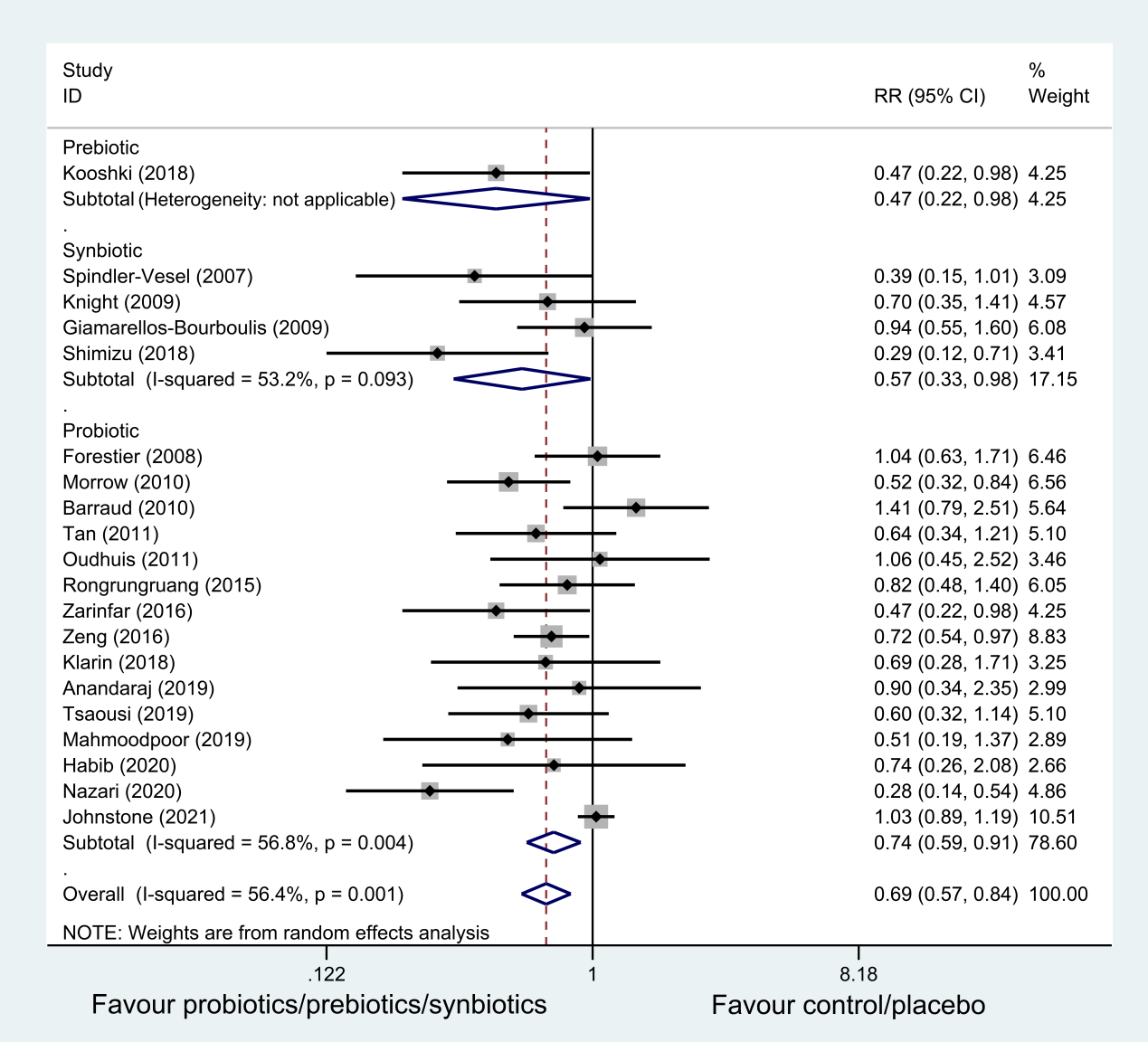


**c.** Forest plot of subgroup analysis by the strain types (prebiotic vs. synbiotic vs. probiotic). RR relative risk, CI confidence interval.


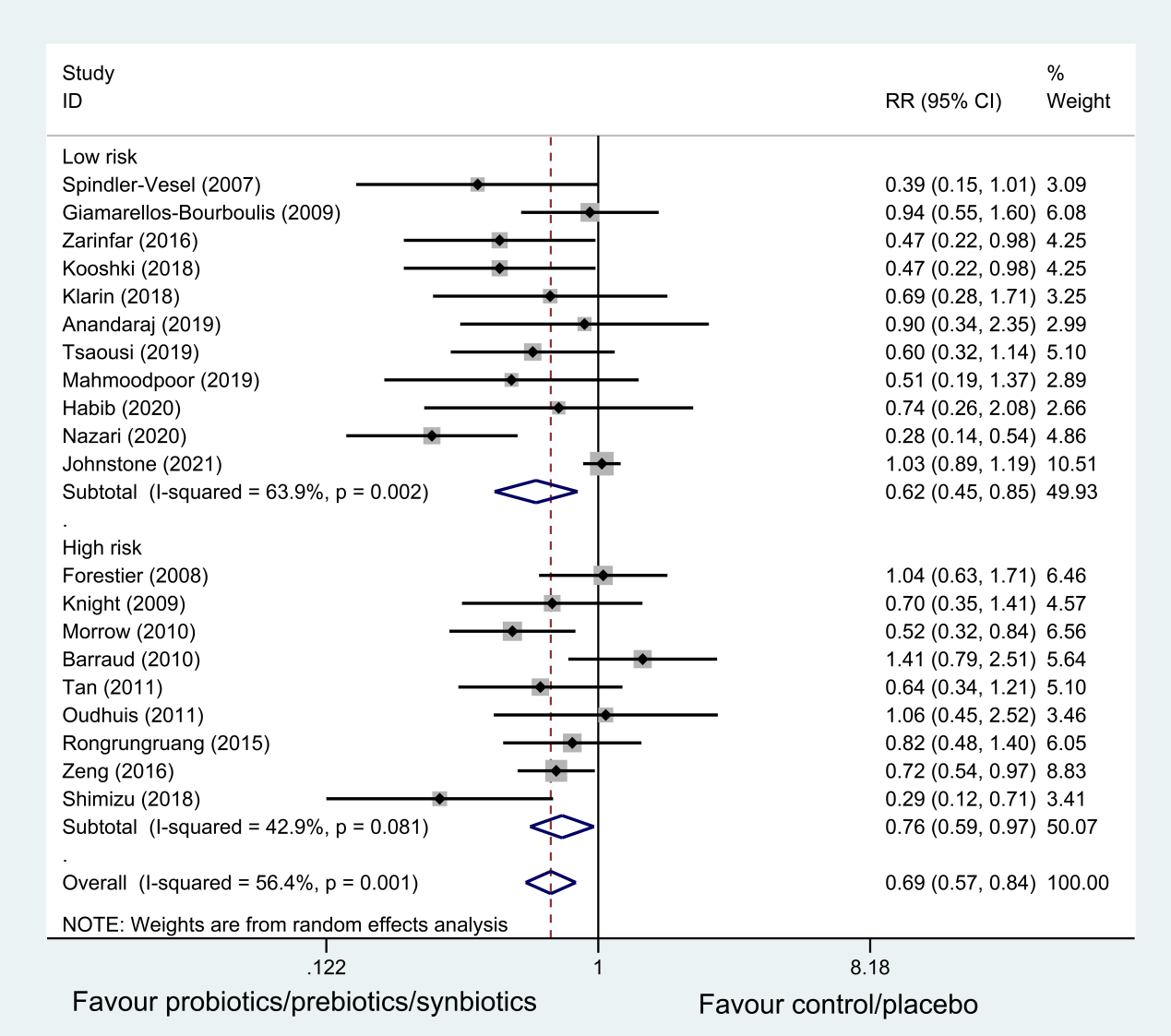


**d．**Forest plot of subgroup analysis by the risk of bias (low risk vs. high risk). RR relative risk, CI confidence interval.


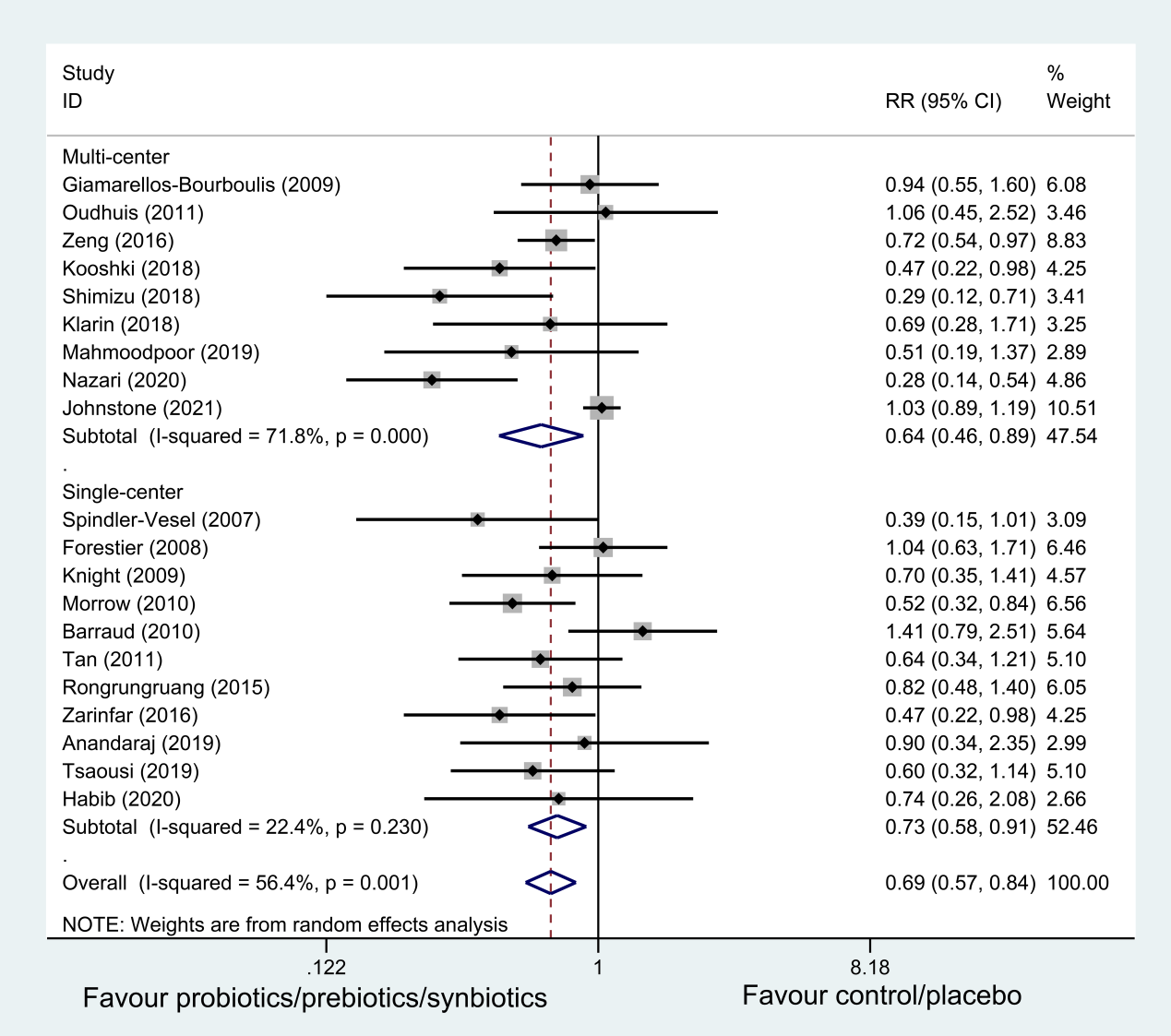


**e.** Forest plot of subgroup analysis by type of the center (multi-center vs. single-center). RR relative risk, CI confidence interval.
